# Additive-Manufactured S53P4@PCL Composite Scaffolds Functionalized with Aptamers and Antibacterial Exosomes for Rapid Bacterial Capture and Killing

**DOI:** 10.3390/jfb17040174

**Published:** 2026-04-01

**Authors:** Chen Zhang, Runyi Lin, Jinchao You, Yaomei Wang, Haopeng Wang, Yixian Ru, Shunxue Xing, Junxiang Wang, Shan Chen

**Affiliations:** 1 School of Materials and Chemistry Engineering, College of Geography and Oceanography, Minjiang University, Fuzhou 350108, China; zhangchen@mju.edu.cn (C.Z.); wangyaomei@stu.mju.edu.cn (Y.W.); xingshunxue@stu.mju.edu.cn (S.X.); 2 School of Pharmaceutical Sciences, Jilin University, Changchun 130021, China; linry25@mails.jlu.edu.cn (R.L.); ruyx24@mails.jlu.edu.cn (Y.R.); 3 School of Public Health, Fujian Medical University, Fuzhou 350108, China; youjinchao@fjmu.edu.cn; 4 CAS Key Laboratory of Design and Assembly of Functional Nanostructures, Fujian Key Laboratory of Nanomaterials, Fujian Institute of Research on the Structure of Matter, Chinese Academy of Sciences, Fuzhou 350002, China; wanghaopeng@fjirsm.ac.cn

**Keywords:** additive manufacturing, S53P4@PCL, bone tissue engineering, nucleic acid aptamers, exosomes

## Abstract

Bone defects remain a significant challenge in bone tissue engineering, driving an urgent need for advanced materials with enhanced therapeutic properties. Additive manufacturing highlights a unique capacity for customization, which enables the precise realization of complex and personalized composite scaffolds. This study innovatively integrates the superior mechanical properties of polycaprolactone (PCL) with the antibacterial characteristics of S53P4 bioactive glass. Utilizing thermal melt extrusion processing and fused deposition modeling (FDM) technology, we fabricated gradient-structured S53P4@PCL composite three-dimensional porous scaffolds with varying doping ratios (5 wt%, 10 wt%, 20 wt%). To further improve the antibacterial efficacy of the scaffold, exosomes (EXO) derived from grouper eggs were functionalized with bacteria-targeting aptamers (APTs), a type of functional DNA capable of binding to bacterial peptidoglycan, and EXO-APT-20%S53P4@PCL was fabricated. The resulting EXO-APT-20%S53P4@PCL scaffold was able to facilitate the targeted capture and subsequent eradication of bacteria. This study pioneers the synergistic integration of aptamer-modified exosomes into 3D composite scaffolds. Our analysis confirmed that the incorporation of APTs enabled targeted bacterial capture, and antibacterial EXO further enhanced the overall bacterial killing capability of the S53P4@PCL scaffolds. The fabrication of porous S53P4@PCL scaffolds through an innovative composite-molding strategy, combined with EXO-APT functionalization, establishes a new paradigm for customized bone repair.

## 1. Introduction

Bone defects remain a major clinical challenge, causing millions of surgical interventions worldwide each year. When the defect size exceeds the critical threshold, the body will lose its intrinsic regenerative capacity, making surgical intervention unavoidable. Currently, autografts, allografts, and xenografts are considered the gold standard for bone defect repair. However, these approaches present significant limitations, such as limited donor availability, risk of immune rejection, and insufficient efficacy in treating critical-sized bone defects [1,2]. Therefore, the development of alternative strategies for effective bone regeneration is of great clinical importance.

With recent advances in biomaterials and tissue engineering, artificial bone scaffolds have emerged as promising alternatives to conventional bone grafts. Among various fabrication techniques, additive manufacturing has attracted considerable attention due to its ability to precisely control scaffold geometry and microarchitecture, enabling the customization of patient-specific implants [3]. Fused deposition modeling (FDM), in particular, is one of the most widely used 3D-printing techniques because of its cost-effectiveness, convenience, and use of standardized printing materials [4]. It has been extensively applied in biomedical engineering, electronics, and aerospace [5,6,7].

Bioactive glass (BG) has been widely investigated for bone tissue engineering due to its excellent osteoconductivity and bioactivity [8]. S53P4, a clinically available bioactive glass originally developed at Åbo Akademi University in Finland, an available bioactive glass with a composition of 53 wt% SiO_2_, 20 wt% CaO, 23 wt% Na_2_O, and 4 wt% P_2_O_5_, is of particular interest because of its unique antibacterial properties. Currently, this material has been applied clinically and is manufactured as a medical-grade product, such as Bonalive. Unlike most BGs, S53P4 has been proven to effectively inhibit bacterial growth, including multidrug-resistant strains [9,10,11]. Its antibacterial activity is attributed to a dual mechanism: (i) the release of Na^+^ ions increases local pH, creating an alkaline environment unfavorable for bacterial survival, and (ii) the elevated concentrations of Na, Ca, Si, and P ions increase osmotic pressure, further suppressing bacterial proliferation [12,13]. Importantly, the biological performance of bioactive glasses can also be influenced by manufacturing processes, and materials with identical nominal compositions may exhibit different physicochemical and biological properties.

Polycaprolactone (PCL) is a biodegradable polyester commonly used as a scaffold matrix owing to its excellent biocompatibility, low toxicity, slow degradation rate, and favorable mechanical properties [14,15]. Its chemical structure allows blending with various polymers without losing its inherent advantages [16]. However, PCL has notable limitations, including hydrophobicity and a slow degradation rate, which hinder fluid infiltration and fail to match the rate of new bone formation [17]. Incorporating bioactive glass and other degradable materials has been reported to improve PCL’s osteogenic and antibacterial performance [18,19,20,21,22,23].

In parallel, bio-functional modifications have been explored to further enhance scaffold performance. Nucleic acid aptamers, single-stranded DNA (ssDNA) or RNA molecules capable of folding into complex secondary or tertiary structures, exhibit high affinity and specificity toward target molecules [24,25]. Compared with antibodies, aptamers offer several advantages, including chemical synthesis, facile modification, low batch-to-batch variability, minimal immunogenicity, and low toxicity [26,27]. Aptamer-based strategies have been successfully applied in bacterial detection and capture. For example, Wenjian et al. developed an electrochemical biosensor for *Salmonella* detection, and Li et al. identified high-affinity aptamers for rapid detection of *Pseudomonas aeruginosa* [28,29].

Exosomes, nanosized extracellular vesicles (30–150 nm) secreted by cells, have also attracted growing interest due to their role in intercellular communication and their potential as antibacterial agents [30,31,32,33]. They are lipid bilayer–enclosed vesicles that carry proteins, nucleic acids, and metabolites capable of modulating immune responses [34,35]. Recent studies have demonstrated the antibacterial potential of exosome-based therapies. For instance, Li et al. designed an exosome-modified bio-heterostructure with potent antibacterial and antitumor effects [36]. Aptamer-functionalized nanovesicles have been widely reported to improve targeting capability and binding specificity in various biomedical applications, including targeted drug delivery and tumor therapy [37,38]. Based on this rationale, the incorporation of aptamers onto exosomes may confer selective bacterial recognition, thereby promoting targeted interaction and enhancing the local antibacterial action of exosomes. Combining exosomes with aptamers could therefore provide a novel strategy for bacterial capture and inhibition.

In this study, we fabricated porous composite scaffolds with gradient compositions using FDM, incorporating S53P4 bioactive glass (5, 10, and 20 wt%) into a PCL to construct S53P4@PCL scaffolds. The scaffolds were systematically characterized for their physicochemical properties, modified with exosomes and aptamers, and evaluated for antibacterial performance. This work aims to develop a multifunctional scaffold with enhanced osteogenic and antibacterial properties, providing a promising candidate for bone defect repair.

## 2. Materials and Methods

### 2.1. Fabrication of S53P4@PCL Scaffolds

PCL granules from SOLVAY (Brussels, Belgium) and S53P4 powders from Kunshan Technology New Materials Co., Ltd. (Kunshan, China) were weighed at mass ratios of 95:5, 90:10, and 80:20. The mixtures were blended in a two-roll die cutter (LN-LT-4, Guangdong Lina Industrial Co., Ltd., Dongguan, China) at 45–50 °C with a roller gap of 0.3–0.7 mm to obtain composites containing 5, 10, and 20 wt% S53P4. The blended materials were flattened, air-dried, and labeled. Dried composites were cut into ~0.5 cm strips, pelletized, and dried again. Filaments suitable for FDM were produced by repeated extrusion using a twin-screw extruder (PloyLab Os, Thermo Fisher Scientific, Bremen, Germany), maintaining a diameter of 1.75 ± 0.05 mm. Cylindrical porous scaffolds (porosity 50%, 15 mm in diameter, 3 mm in height) were designed using Snapmaker Luban 4.7.2 with the following printing parameters: a nozzle temperature of 85 °C and a bed temperature of 40 °C. Scaffolds with 0, 5, 10, and 20 wt% S53P4 (denoted as PCL, 5%S53P4@PCL, 10%S53P4@PCL, and 20%S53P4@PCL) were fabricated via FDM 3D printing.

### 2.2. Morphological Characteristics

The macroscopic morphology of S53P4 was visually examined. For each group (n = 3), the diameter and height of pure PCL and S53P4@PCL scaffolds were measured with a vernier caliper (Shanghai Tools Company, Shanghai, China) and weighed with an analytical balance (Kunshan Scientific Instrument Company, Kunshan, China).

Microscopic morphology of the powder and scaffold was characterized by scanning electron microscopy (SEM, Hitachi E-1045, Tokyo, Japan) at magnifications of 1000×, 2000×, 5000×, and 10,000×. SEM results were used to observe and analyze the changes in the scaffolds incorporating different proportions of BG S53P4.

### 2.3. Fourier Transform Infrared (FTIR) Spectroscopy

The PCL composite materials containing 5 wt%, 10 wt% and 20 wt% BG S53P4 were pressed into sheets with a diameter of 20 mm and a thickness of 1 mm for use. Pure PCL and S53P4@PCL composites (5, 10, and 20 wt%) were analyzed by FTIR (Nicolet iS20, Thermo Fisher Scientific, Waltham, MA, USA) to determine their chemical structures.

### 2.4. Contact Angle Measurement

In order to observe the hydrophilicity of different composite materials, the previously prepared PCL and S53P4@PCL composites (5, 10, and 20 wt%) sheets were tested. The water contact angle was measured using a contact angle goniometer (JC2000D1, Zhongchen Digital Technic, Shanghai, China).

### 2.5. Melt Flow Rate (MFR)

We are conducting an investigation to determine whether the composite materials with different ratios of S53P4@PCL can meet the flow characteristics required for additive manufacturing. The MFR of pure PCL and S53P4@PCL composites (5, 10, and 20 wt%) was measured according to ASTM D1238 using a melt flow rate tester (XNR-400A, Dahua Instruments, Chengde, China) at 125 °C.

### 2.6. Dynamic Rheology

Assessment of the rheological properties of S53P4@PCL composites with different proportions was carried out. Pure PCL and S53P4@PCL composites were molded into circular disks (15 mm in diameter, 2 mm in thickness) using a plate vulcanizer. Temperature-sweep tests were conducted from 75 °C to 120 °C on a polymer rotating rheometer system (DHR-2, TA Instruments; Newcastle, DE, USA).

### 2.7. Thermal Analysis

Thermal behavior was analyzed using granulated samples under a constant heating rate. PCL and S53P4@PCL composite samples were heated from −80 °C to 100 °C, cooled to −80 °C, and reheated to 100 °C to eliminate thermal history interference.

### 2.8. In Vitro Degradation Test of S53P4@PCL Scaffolds

Three scaffolds were collected from each of four groups of pure PCL scaffolds and composite scaffolds containing 5%, 10%, and 20% S53P4@PCL. After weighing and recording, all scaffolds were immersed in 75% ethanol and ultrasonically treated for 5 min, rinsed with sterile water, and then air-dried in a cleanroom and UV-exposed for 20 min. The four scaffolds and 20 mL of PBS simulated body fluid were placed in a 50 mL centrifuge tube and incubated at 37 °C for 32 days. The scaffolds were then removed, oven-dried until their mass remained constant, and weighed and recorded.

(1) $$∆\left(mt\%\right)=\left[\frac{M_{0}-M_{t}}{M_{0}}\right]\times 100\%$$

∆ is the in vitro degradation rate, M_0_ is the mass of each scaffold before degradation, and M_t_ is the weight of the scaffold after 32 days of incubation and degradation in simulated body fluid.

### 2.9. Recruitment of APTs to Staphylococcus Aureus

To enhance the antibacterial efficiency of the composite scaffold, the APTs we selected (as shown in Table 1) were employed to recruit bacteria. In order to verify whether the APTs have the ability to recruit bacteria, PCL sheets (1 cm × 1 cm) were modified using 0.5 mg/mL dopamine hydrochloride at alkaline pH for 4 h to obtain PDA-PCL. The APTs were immobilized on PCL and PDA-PCL, forming APT-PCL and APT-PDA-PCL, respectively. Each sample was incubated in 5 mL of *S. aureus* suspension (OD_600_ = 0.1) for 1 h. The OD_600_ of the remaining suspension was measured, and Gram staining was performed on the suspension to evaluate the APTs’ ability to attract *Staphylococcus aureus* in a system.

### 2.10. Preparation of EXO-APT-20%S53P4@PCL Scaffolds

The aptamer sequences used in this study were synthesized and purified by Sangon Biotech (Shanghai, China) with a cholesterol molecule covalently attached to the 5′-end via a phosphoramidite linker. This cholesterol modification exploits the hydrophobic nature of cholesterol, which facilitates its spontaneous insertion into the phospholipid bilayer membrane of exosomes through hydrophobic interactions. The cholesterol-modified aptamers enable stable anchoring onto the exosome surface without disrupting membrane integrity.

The selected APTs have been shown to have a certain bacterial recruitment effect. Because APTs are single-stranded DNA and are easily chemically modified, a cholesterol molecule was added to the end of the aptamer sequence, allowing the aptamer to be inserted into the phospholipid bilayer of the exosome. The modified APT sequences are listed in Table 1. After this, EXO-APT-20%S53P4@PCL scaffolds were prepared.

Firstly, exosomes are isolated from grouper eggs to prepare a stock solution. Fresh grouper (*Epinephelus*) oocytes were collected and immediately transferred into ice-cold PBS containing protease inhibitors. Samples were transported and stored on ice, then washed three times with pre-chilled PBS to remove mucus and debris. Tissues were homogenized in PBS (*w*/*v* = 1:5). After differential centrifugation to remove cell debris, exosomes were isolated by ultracentrifugation and purified via density gradient centrifugation. The final exosome stock was diluted to 0.3 μg/μL in PBS for further use. Morphological observation by TEM. Grouper egg exosomes (5–10 μL, pre-fixed with 2.5% glutaraldehyde) were dropped onto copper grids and adsorbed at room temperature for ~5 min. Excess liquid was carefully blotted with filter paper, and the grids were negatively stained with 10 μL of saturated uranyl acetate solution for 1 min at room temperature. After removing the staining solution, the grids were rinsed twice with ddH_2_O (5 min each), air-dried at room temperature, and subsequently examined using a transmission electron microscope at an accelerating voltage of 80 kV.

Furthermore, to further characterize the protein markers of the isolated vesicles, Western blot analysis was performed. Grouper egg exosomes were lysed in RIPA buffer containing PMSF on ice for 20 min and centrifuged at 12,000 rpm for 20 min at 4 °C to collect the supernatant. Protein concentrations were determined using a BCA protein assay kit. Equal amounts of protein were denatured with loading buffer at 100 °C for 5 min and separated by SDS–PAGE, followed by transfer onto PVDF membranes. After blocking with 5% BSA for 2 h at room temperature, membranes were incubated overnight at 4 °C with primary antibodies against TSG101 (1:2000), CD63 (1:5000), and Calnexin (1:5000). Membranes were then incubated with HRP-conjugated secondary antibodies and visualized using an enhanced chemiluminescence (ECL) detection system.

Subsequently, cholesterol-modified aptamers were mixed with the exosomes to form an EXO-APT complex. The APTs were reconstituted to 0.1 nmol/μL in buffer. EXO-APT complexes were prepared by mixing exosomes (0.3 μg/μL) with APTs to final concentrations of 50 and 100 nM in PBS (10 mL total volume), followed by incubation for 4 h at 4 °C. Particle size was measured at 1, 2, and 4 h to assess binding efficiency.

**Figure 1:**
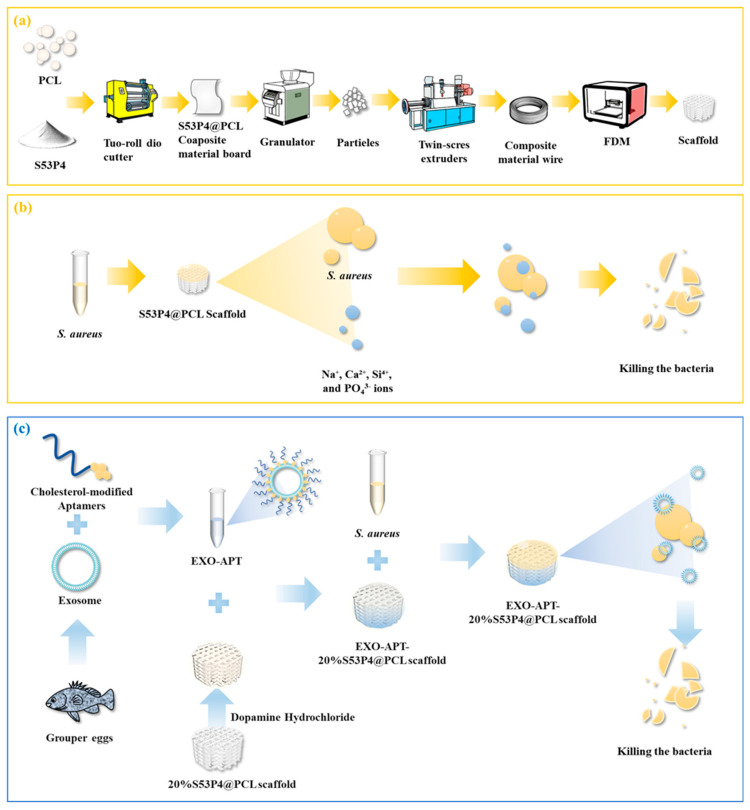
Schematic illustration depicting the construction of antibacterial scaffolds. (**a**) Preparation of 3D-Printed PCL, S53P4@PCL scaffolds. (**b**) Antibacterial mechanism of S53P4@PCL scaffolds. (**c**) Preparation and antibacterial mechanism of EXO-APT-20%S53P4@PCL scaffold.

Finally, the scaffold is functionalized (as shown in Figure 1c). S53P4@PCL scaffolds with 20 wt% S53P4 (optimized based on antibacterial screening) were sterilized, immersed in 0.5 mg/mL dopamine solution for 4 h, and air-dried under sterile conditions. EXO–APT complexes with 100 nM APTs (the concentration yielding maximal particle size) were prepared in 2 mL solution. Scaffolds were incubated in this solution for 12 h to complete surface functionalization.

### 2.11. Antibacterial Performance Evaluation

The S53P4@PCL scaffold releases Na^+^, Ca^2+^, Si^4+^, and PO_4_^3−^ ions, changing the physical and chemical properties of the surrounding environment to achieve antibacterial effects. Furthermore, the EXO-APT-functionalized scaffolds introduce an additional antibacterial mechanism, in which APTs enable the selective recognition and capture of bacteria, while exosomes subsequently exert bactericidal effects, thereby enhancing the overall antibacterial functionality of the scaffold. This study will evaluate the antibacterial properties of the scaffold using the Absorption Method and Agar Diffusion (Inhibition Zone) Assay [39,40,41].

The Absorption Method refers to ASTM E2149. Scaffolds including PCL (control), 5% S53P4@PCL, 10%S53P4@PCL, 20%S53P4@PCL, and EXO-APT-20%S53P4@PCL were sterilized by ethanol immersion, ultrasonication, sterile water rinse, and UV exposure. Each scaffold was placed in a 50 mL tube cap and inoculated with 0.2 mL of *S. aureus* suspension (OD_600_ = 0.1). After 24 h of incubation at 37 °C, 20 mL LB medium was added to elute adherent bacteria. OD_600_ was measured, and the inhibition rate (R) was calculated as follows:

(2) $$R=\frac{\left(A-B\right)}{A}\times 100\%$$

where A and B are the OD values of the control and test groups, respectively.

Additionally, 1 mL of *S. aureus* was inoculated into fresh LB medium and cultured at 37 ± 1 °C for 24 h to observe bacterial growth.

During the Agar Diffusion (Inhibition Zone) Assay, PCL, 5%S53P4@PCL, 10%S53P4@PCL, 20%S53P4@PCL, and EXO-APT-20%S53P4@PCL scaffolds were placed on LB agar plates seeded with *S. aureus* and incubated for 24 h at 37 °C. Inhibition zones were observed and recorded.

### 2.12. Biocompatibility Experiment of S53P4@PCL

NIH3T3 cells were seeded into 96-well plates at a density of 5000 cells per well in DMEM supplemented with 10% FBS and 1% penicillin–streptomycin, and cultured at 37 °C with 5% CO_2_ for 24 h. Meanwhile, extraction media were prepared by immersing 200 mg of 5%, 10%, and 20% S53P4@PCL in 10 mL of complete culture medium within 15 mL centrifuge tubes, and incubating at 37 °C for 24 h. The conditioned media were subsequently filtered through 0.22 μm membrane filters to remove particulates and ensure sterility. The original cell culture medium was then replaced with the corresponding filtered extraction medium, and cells were incubated for a further 24 h. Cell viability was assessed using the CCK-8 assay according to the manufacturer’s instructions, with absorbance measured at 450 nm. All experiments were conducted in triplicate (n = 3), and results are expressed as mean ± standard deviation (SD).

## 3. Results

### 3.1. Morphology and Structure Observation

The entire experimental design is shown in Figure 1, including the composite material to scaffold preparation, the functionalization of the scaffold with exosomes and aptamers, and the overall antibacterial mechanism.

**Figure 2:**
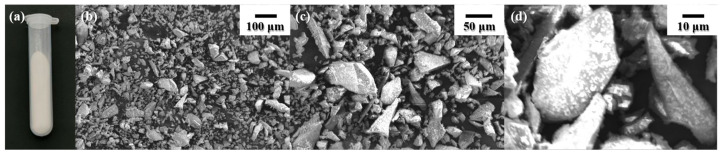
Morphology of S53P4 powder. (**a**) Macrostructure of S53P4; (**b**) SEM of S53P4 powder (200×); (**c**) SEM of S53P4 powder (500×); (**d**) SEM of S53P4 powder (1000×).

S53P4 appeared as a fine, uniform white powder at room temperature. SEM images at various magnifications are shown in Figure 2. S53P4 powder has an irregular crystal structure, and the diameter of the crystal is generally between 5 and 30 μm.

**Figure 3:**
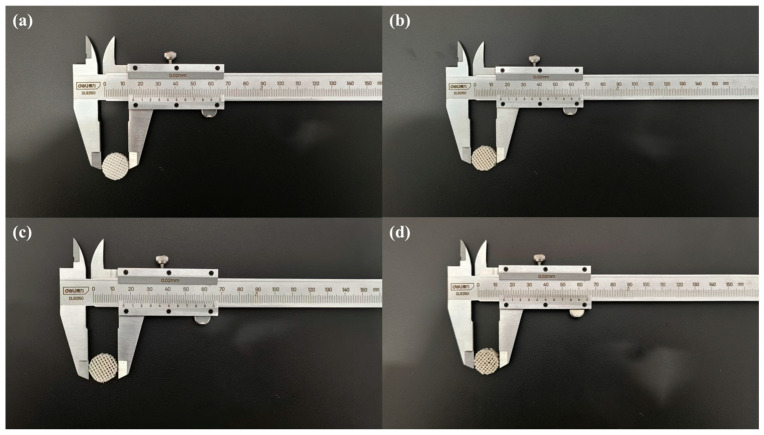
Morphology of S53P4@PCL scaffolds. (**a**) Pure PCL scaffolds; (**b**) 5%S53P4@PCL scaffolds; (**c**) 10%S53P4@PCL scaffolds; (**d**) 20%S53P4@PCL scaffolds.

As shown in Figure 3, the fabricated S53P4@PCL scaffolds exhibited a porous cylindrical structure with a diameter of approximately 15 mm and a height of ~4.2 mm. Pure PCL scaffolds were translucent white, whereas increasing S53P4 content led to an opaque appearance.

Each group of scaffolds was evaluated by measuring three samples to calculate the mean and standard deviation. As shown in Table S1, the average weight of S53P4@PCL scaffolds was approximately 0.42 g. The standard deviations of weight, diameter, and height in all groups were less than 0.5, indicating good reproducibility in scaffold fabrication.

### 3.2. Contact Angle

**Figure 4:**
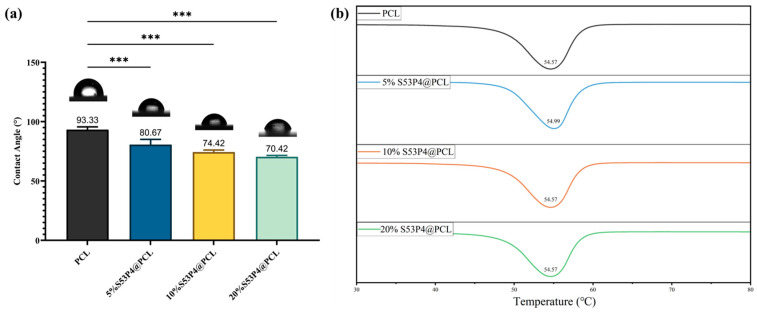
Physicochemical characterization of composites with different S53P4 contents. (**a**) Contact angle test results. Data is shown as mean ± SD (n = 3). One-way ANOVA. *** *p*  <  0.0001. (**b**) The DSC curve of S53P4@PCL composites with different S53P4 content.

Upon implantation, the material surface rapidly adsorbs proteins due to the immune response. Hydrophilic surfaces tend to reduce protein adsorption, thereby potentially mitigating inflammatory reactions [42]. As shown in Figure 4a, the contact angle decreased with increasing S53P4 content. The 20%S53P4 sample showed the greatest reduction, from 93.33° (pure PCL) to 70.42°, indicating enhanced hydrophilicity. These findings suggest that the incorporation of S53P4 effectively enhances the surface wettability and may reduce inflammatory responses.

### 3.3. Thermal Properties

As shown in Figure 4b, the crystallization temperature (Tc) of the pure PCL group was 54.57 °C. The addition of 5% S53P4 significantly increased Tc to 54.99 °C. However, the Tc of the 10% and 20% S53P4 groups returned to levels similar to those of pure PCL. This trend suggests that the interaction between different concentrations of S53P4 and PCL caused the crystallization temperature to first increase and then return to the level of pure PCL.

### 3.4. Melt Flow Rate (MFR) Analysis

**Figure 5:**
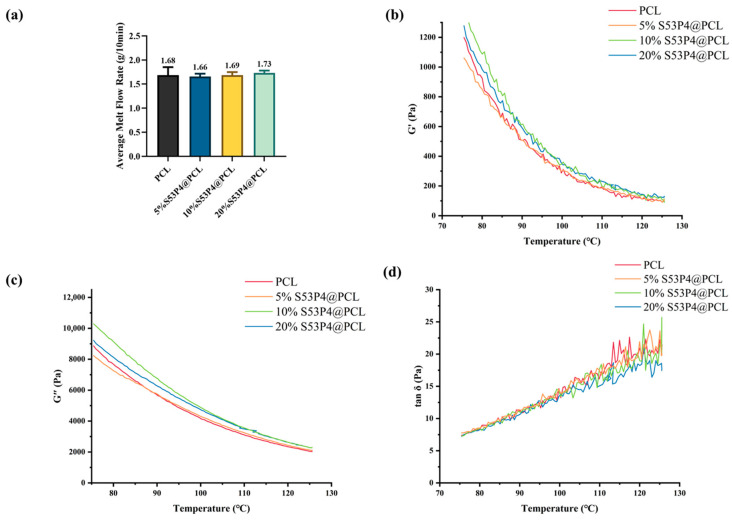
Average melt flow rate and dynamic rheological behavior of the composites. (**a**) Average melt flow rate of the composites; (**b**) storage modulus (G′); (**c**) loss modulus (G″); and (**d**) loss tangent (tan δ).

The melt flow rate is a key indicator of material processability during extrusion-based additive manufacturing. As shown in Figure 5a, the addition of S53P4 did not significantly affect the melt flow rate of PCL, and the composite maintained suitable flow properties for printing.

### 3.5. Dynamic Rheological Behavior

The ratio of storage modulus (G′) to loss modulus (G″), represented by tanδ, influences the solidification and interlayer adhesion during cooling. Excessively rapid solidification may weaken interlayer bonding, while too slow solidification can result in deformation during printing. As shown in Figure 5b–d, the tanδ values of PCL mixed with various concentrations of S53P4 differed to varying degrees, but all exhibited suitable rheological behavior for additive manufacturing.

### 3.6. In Vitro Degradation of S53P4@PCL Scaffolds

The in vitro degradation of the four scaffold groups over a 32-day period is summarized in Table S2. The average degradation rates for PCL, 5%S53P4@PCL, 10%S53P4@PCL, and 20%S53P4@PCL were calculated to be 0.3996%, 0.7364%, 0.7577%, and 1.42%, respectively. A progressive increase in degradation rate was observed with higher S53P4 content, indicating that the incorporation of bioactive glass significantly accelerated the degradation of the composite scaffolds. These results suggest that adjusting the S53P4 content provides an effective strategy for optimizing scaffold degradation to better match bone regeneration requirements.

**Figure 6:**
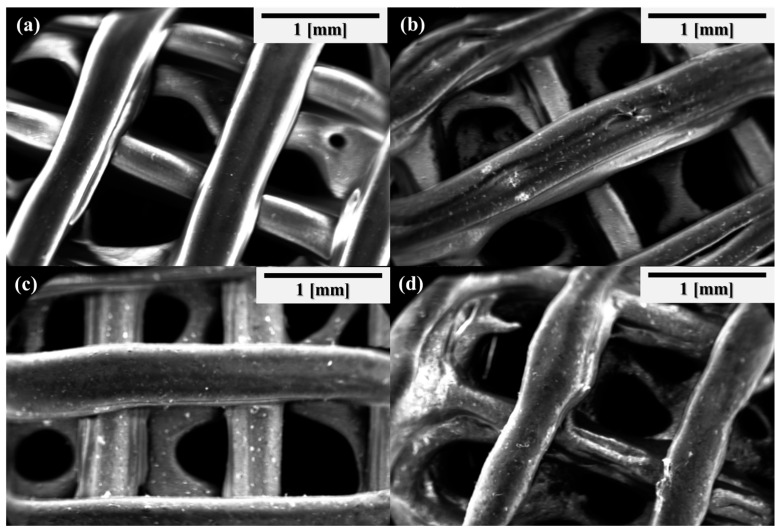
SEM images of composite scaffolds after in vitro degradation. (**a**) PCL scaffolds (**b**) 5%S53P4@PCL scaffolds; (**c**) 10%S53P4@PCL scaffolds; (**d**) 20%S53P4@PCL scaffolds.

The scaffolds were further observed using SEM following degradation. As shown in Figure 6, distinct surface morphological changes were observed in all samples compared to non-degraded scaffolds. Significantly, scaffolds containing S53P4 exhibited rougher surface textures, indicating that the presence of bioactive glass effectively promoted the degradation process.

### 3.7. Recruitment of Staphylococcus Aureus via Bacterial Peptidoglycan-Targeting Aptamers

PDA is a biocompatible and versatile coating material. DNA can be adsorbed onto PDA by adjusting its pH [43]. Therefore, the method of linking DNA to materials through PDA can verify the adsorption effect of DNA on bacteria.

EDS was employed to analyze the elemental composition of scaffold surfaces by detecting characteristic X-rays emitted under electron beam excitation in vacuum conditions. As shown in Figure S1, scaffolds containing S53P4 exhibited new peaks compared to pure PCL, corresponding to elements such as SiO_2_, CaO, and Na_2_O from the bioactive glass. The PDA-modified scaffolds showed additional characteristic peaks due to the PDA modified scaffold derived from Dopamine hydrochloride, and the intensity of the other characteristic peaks was lower than that of 20%S53P4@PCL, confirming the surface modification. Therefore, PDA can be successfully covered on the surface of the composite material.

PCL sheets, APT-PCL sheets, and APT-PDA-PCL sheets were each incubated in *S. aureus* suspension (OD_600_ = 0.1) for 1 h. After removing the sheets, the OD_600_ of the remaining suspension was measured. After significance analysis (Figure S2), no significant change in bacterial density was observed after treatment between PCL and PDA-PCL films. However, a marked increase in OD_600_ was detected in the group treated with APT-PDA-PCL, indicating a notable difference compared to the other two groups. Gram staining results also proved that the number of bacteria in the remaining bacterial solution of the APT-PDA-PCL treatment group was smaller. These results suggest that the aptamers used in this study possess a recruitment effect toward *S. aureus*, and the influence of PDA alone can be excluded.

### 3.8. Morphological Characteristics of Exosomes and EXO-APT Complexes

**Figure 7:**
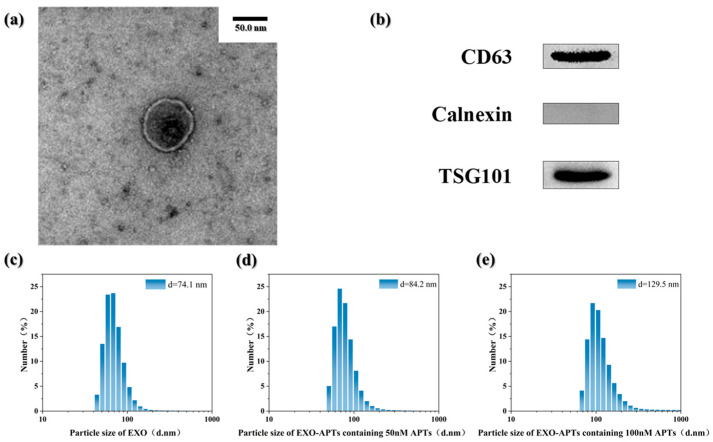
Morphological characteristics of exosomes and EXO-APT complexes. (**a**) TEM image of grouper eggs exosomes. (**b**) Exosome characteristic proteins of grouper egg exosomes. (**c**) Particle size of EXO by Malvern particle size analyzer. (**d**,**e**) Particle size of EXO-APTs containing APTs of different densities by Malvern particle size analyzer.

Grouper egg exosomes were isolated by ultracentrifugation. Transmission electron microscopy (TEM) results (Figure 7a) show that the purified grouper egg exosomes are uniform in morphology, with a distinct double-layer membrane structure, consistent with the microscopic identification characteristics of exosomes.

As shown in Figure 7b, Western blot analysis demonstrated the presence of the exosomal markers CD63 and TSG101 in the isolated vesicles, whereas the endoplasmic reticulum marker Calnexin was absent, suggesting the successful isolation of exosomes.

Particle size analysis by Malvern particle size analyzer (Zetasizer Nano ZS90, Malvern Instruments Ltd., Malvern, UK) showed that the average diameter of isolated exosomes was approximately 74.1 nm (Figure 7c), falling within the established exosome size range (30–150 nm). As shown in Figure 7d,e, the particle sizes of EXO-APT complexes varied with aptamer concentration. An increase in particle size reflects a greater number of aptamers bound to the exosome surface, indicating more efficient complex formation. The particle size reached its maximum at an APT concentration of 100 nM, suggesting that this concentration provides optimal conditions for EXO-APT binding. Collectively, these morphological and size distribution characteristics, consistent with the MISEV guidelines, confirm the successful isolation of exosomes from grouper eggs.

### 3.9. Characterization of EXO-APT-20%S53P4@PCL Composite Scaffolds

**Figure 8:**
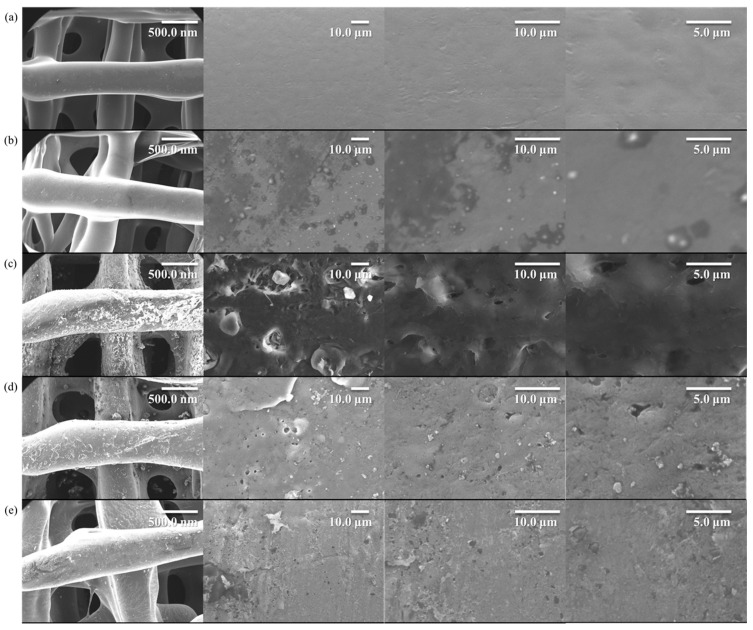
SEM images of different scaffolds at various magnifications. (**a**) Pure PCL, (**b**) 20%S53P4, (**c**) PDA-modified 20%S53P4@PCL, (**d**) EXO-modified 20%S53P4@PCL, and (**e**) EXO–APT- 20%S53P4@PCL.

Scanning electron microscopy (SEM) images of the scaffolds are shown in Figure 8. Panel a represents PCL, while Panel b shows 20%S53P4@PCL. Panels c to e correspond to scaffolds modified with dopamine (c), dopamine plus exosomes (d), and dopamine plus EXO-APT complexes (e), respectively. In Panel b, the incorporation of 20% S53P4@PCL slightly altered the scaffold surface morphology. Panel c exhibits a rougher surface with visible particulate structures, indicating successful dopamine hydrochloride modification. As shown in Panels d and e, the number and size of surface particles were markedly reduced, suggesting that the exosome and EXO-APT complexes had effectively coated the scaffold surface and the EXO-APT-20%S53P4@PCL composite scaffolds had been successfully prepared.

### 3.10. Antibacterial Activity of S53P4@PCL Scaffolds

Postoperative infections in orthopedic surgery are predominantly caused by Gram-positive cocci, particularly *Staphylococcus aureus*, while Gram-negative bacilli such as Escherichia coli and Pseudomonas aeruginosa may also be involved in specific clinical settings, including trauma-related or nosocomial infections. Anaerobes, fungi, and Mycobacterium species are less common but have also been reported [44,45]. Among these, *Staphylococcus aureus* is considered the most predominant and clinically significant pathogen. Therefore, antibacterial tests were performed by co-culturing *S. aureus* with both functionalized and unfunctionalized composite scaffolds.

**Figure 9:**
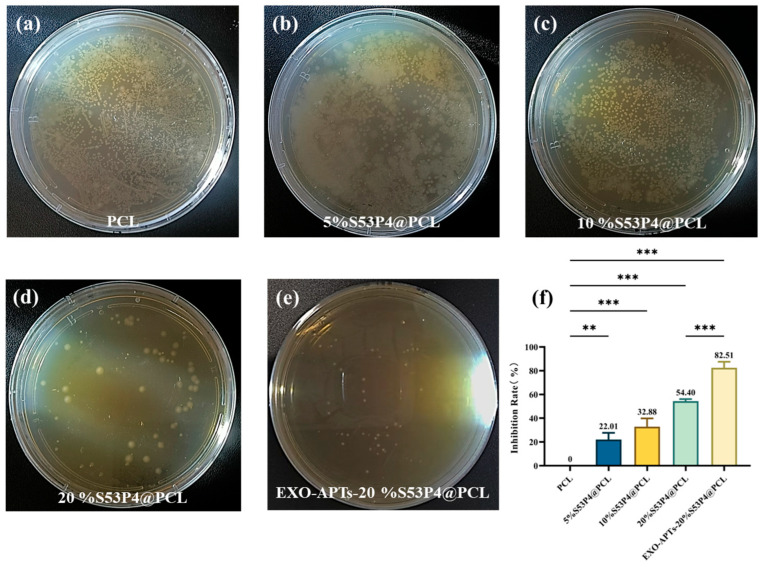
Antibacterial activity of S53P4@PCL scaffolds. (**a**–**e**) Dilution coating result chart. (**f**) Inhibition rate of scaffolds. Data are shown as mean  ±  SD. n  =  3 samples per group. One-way ANOVA. ** *p*  <  0.01; *** *p*  <  0.001.

A bacterial suspension of *S. aureus* was prepared with an initial OD_600_ of 0.1. The control group (pure PCL) and experimental groups (5%S53P4@PCL, 10%S53P4@PCL, 20%S53P4@PCL, and EXO-APT-20%S53P4@PCL) were co-incubated with the bacterial suspension for 24 h. After repeating the process three times, record the OD600 of the bacterial suspension after incubation and calculate the inhibition rate, as shown in Table S3. Subsequently, the bacterial suspensions were plated on solid agar medium and incubated for another 24 h. The results are shown in Figure 9a–e, and the statistical analysis of the antibacterial rate is shown in Figure 9f.

The data demonstrated a gradual decrease in bacterial density with increasing S53P4 content, indicating that the percentage of S53P4 influences the antibacterial effect. In particular, the scaffold modified with EXO-APTs exhibited enhanced antibacterial performance compared to unmodified scaffolds. The diameters of the inhibition zones of the five groups of scaffolds were measured (Figure S3) and analyzed. The inhibition zone test results, shown in Figure S3b, exhibited the same trend as the inhibition rate, indicating that antibacterial activity generally increased with increasing S53P4 content and EXO-APT modification.

The improved antibacterial activity of the EXO-APT-20%S53P4@PCL is mainly attributed to the synergistic effects of aptamers and exosomes. Aptamers facilitate the selective recognition and capture of bacteria, while exosomes contribute to antibacterial effects through their bioactive cargo. This combination enhances the antibacterial ability of scaffolds.

### 3.11. Biocompatibility Experiment of S53P4@PCL

**Figure 10:**
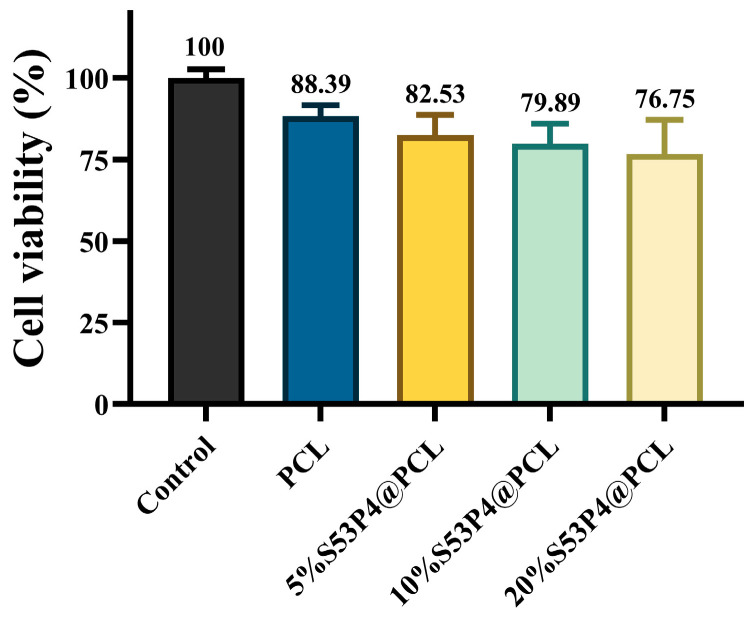
Viability of NIH3T3 cells was assessed by CCK-8 assay, after treatment for 24 h with different S53P4@PCL composite materials.

Cell viability of NIH3T3 cells cultured with PCL and S53P4@PCL composite materials with different S53P4 contents was evaluated by the CCK-8 assay. As shown in Figure 10, all groups exhibited cell viability above 70%, indicating no cytotoxicity according to the GB/T 16886.5-2017 Chinese Standard [14].

## 4. Discussion

In this study, S53P4@PCL composite scaffolds with different concentration gradients were fabricated. The results demonstrated that the incorporation of S53P4 significantly enhanced the antibacterial activity, mechanical properties, and hydrophilicity of the scaffolds, while maintaining appropriate degradation behavior. The scaffolds with 20%S53P4 exhibited outstanding antibacterial performance, which were functionalized with EXO-APTs to further enhance the antibacterial ability of the scaffolds. The integration of EXO-APTs imparts a novel antibacterial capability to the scaffold by leveraging APTs for targeted bacterial recruitment and marine-derived EXO for bacterial killing. As a result, combined with the inherent antimicrobial properties of S53P4, this strategy creates a dual-functional surface that substantially enhances bacterial clearance of the composite scaffold.

Currently, research regarding S53P4@PCL-based composite scaffolds remains limited. Notably, Yu and Fan et al. utilized 3D printing to functionalize the established clinical material, bioactive glass (BG) S53P4, incorporating it with PCL and bone morphogenetic protein-2 (BMP-2) to develop customized bioscaffolds. Their study explored improved strategies for mastoid obliteration and external auditory canal reconstruction [46]. Compared with the previous study, this study provides a more comprehensive physicochemical characterization of the composite materials. We introduced a novel porous structure scaffold and employed diversified methodologies to validate antibacterial efficacy. Studies have shown that exosomes contain cytokines with antimicrobial properties, as well as innate immune signaling molecules, enabling effective antibacterial action and contributing to a strengthened host defense against viral and bacterial challenges [46]. Aptamers, defined as short single-stranded DNA or RNA oligonucleotides, have been employed in biosensing platforms for bacteria detection due to their high affinity and selectivity [47,48]. The antibacterial mechanisms of S53P4 and EXO-APTs are fundamentally different yet complementary. S53P4 exerts its antimicrobial effects through physicochemical mechanisms. During degradation, it releases ions such as Na^+^, Ca^2+^, Si^4+^, and PO_4_^3−^, which increase local osmotic pressure. Simultaneously, the exchange of Na^+^ with H^+^ in the surrounding medium elevates the local pH, creating an alkaline environment unfavorable for bacterial survival. Additionally, calcium ions can interfere with bacterial cell membrane function. This physicochemical mechanism is broad-spectrum but non-specific, affecting all bacteria in the vicinity. In contrast, the EXO-APT system provides a targeted, multi-step biological antimicrobial strategy. The cholesterol-anchored aptamers displayed on the exosome surface specifically recognize and bind to bacterial peptidoglycan, actively recruiting and concentrating pathogens onto the scaffold surface. This targeted capture mechanism ensures that bacteria are brought into close proximity with the scaffold, thereby enhancing the contact efficiency between local bacteria and antimicrobial factors. Meanwhile, exosomes derived from grouper eggs themselves contain antimicrobial peptides, cytokines, and innate immune signaling molecules. These components can both directly disrupt bacterial membrane structures and activate the host’s own defense pathways. This exosome-mediated bactericidal effect is biologically driven and may work synergistically with the scaffold’s physicochemical antimicrobial properties. The integration of these two distinct mechanisms ultimately creates a synergistic antibacterial effect: after concentrating bacteria near the scaffold surface through aptamer-mediated capture, the EXO-APT system not only enhances bacterial exposure to exosome-derived antimicrobial factors but also strengthens their interaction with ions released from S53P4. This dual-action mechanism is the core reason for the significantly higher bacterial inhibition rates observed for EXO-APT-20%S53P4@PCL compared to scaffolds with S53P4 alone. For instance, Murat Kavruk et al. generated aptamers targeting specifically *S. pneumoniae* as a whole pathogen within complex bacterial mixtures. By biomimetically modifying the surface of magnetic particles with polydopamine, they successfully immobilized these aptamers to recognize and sequester *S. pneumoniae* from both PBS and blood samples [49]. Yibiao Wang et al. developed a double-bundle DNA tetrahedron nanocarrier for intratumor bacteria-targeted berberine delivery, offering a promising strategy to modulate the bacteria-associated tumor microenvironment [50]. In this study, the 20%S53P4@PCL composite scaffold was treated with polydopamine to modify EXO-APTs on the surface of the composite scaffold, thus fabricating the EXO-APT-20%S53P4@PCL scaffold. To comprehensively evaluate its antibacterial performance, both inhibition zone and bacterial adsorption assays were employed, as these methods have been used in previous studies [39,40,41].

In summary, the S53P4@PCL composite porous scaffold developed in this study shows favorable hydrophilicity, mechanical performance, antibacterial efficacy, and a controllable degradation rate. After EXO-APT equipment, the EXO-APT-20%S53P4@PCL scaffold yielded a synergistic antimicrobial effect, resulting in significantly higher bacterial eradication rates compared to the scaffold alone. But some limitations remain. The present study focused on in vitro evaluation, and in vivo performance has yet to be investigated. In addition, the antibacterial assays were limited to *Staphylococcus aureus*; thus, future studies should include other clinically relevant pathogens. Future work will therefore focus on in vivo animal models to validate the translational potential of the S53P4@PCL scaffolds in bone regeneration.

## 5. Conclusions

In this study, porous polycaprolactone (PCL) scaffolds composited with bioactive glass S53P4 in different gradient distributions were successfully fabricated and systematically characterized in terms of physicochemical properties, mechanical performance, in vitro degradation, surface modification, and antibacterial activity. Melt flow rate measurements, dynamic rheological analysis, and thermal property evaluations demonstrated that the incorporation of S53P4 significantly enhanced the physical properties of PCL scaffolds, improving rheological behavior and thermal stability. These findings suggest we have uncovered a promising strategy for tuning the physical performance of bio-scaffold materials.

Contact angle measurements and in vitro degradation studies revealed that the presence of S53P4 accelerated scaffold degradation and improved hydrophilicity. Antibacterial assays showed that increasing S53P4 content enhanced the antibacterial efficacy against *Staphylococcus aureus*, particularly when combined with aptamer-targeted bacterial capture and exosome-mediated bacterial killing. This hybrid functionalization strategy not only ensures effective pathogen eradication but also supports a favorable regenerative niche by suppressing infection-related interference.

## Figures and Tables

**Table 1:** APT sequence.

Oligo Name	Sequence (5′–3′)
a	Cholesterol-TTTGGGACAGGGAGTGCGCTGCTCCCCTTTTCGC

## Data Availability

The original contributions presented in this study are included in the article/Supplementary Material. Further inquiries can be directed to the corresponding authors.

## Supplementary Materials

The following supporting information can be downloaded at: https://www.mdpi.com/article/10.3390/jfb17040174/s1, Table S1: Dimensional parameters of PCL@S53P4 scaffolds; Table S2: In vitro degradation of PCL@S53P4 scaffolds; Table S3: Co-culture of Scaffolds with *S. aureus*; Figure S1: EDS of PDA-20%S53P4@PCL scaffold; Figure S2: OD_600_ of the remaining suspension was measured after removing the sheets; Figure S3: Inhibition zone of scaffolds.

## Abbreviations

BG	Bioactive glass
PCL	Polycaprolactone
EXO	Exosome
APT	Aptamer
FDM	Fused Deposition Modeling
FMR	Melt Flow Rate
SEM	Scanning Electron Microscope
TEM	Transmission Electron Microscope
EDS	Energy-Dispersive X-Ray Spectroscopy
DMEM	Dulbecco’s Modified Eagle’s Medium
FBS	Fetal bovine serum
CCK-8	Cell Counting Kit-8
