# Supplementary material for: Additive-Manufactured S53P4@PCL Composite Scaffolds Functionalized with Aptamers and Antibacterial Exosomes for Rapid Bacterial Capture and Killing
Source: J Funct Biomater. 2026 Apr 1;17(4):174. doi: 10.3390/jfb17040174 (PMC13117607; doi:10.3390/jfb17040174)
Supplement: Supplementary file 1 [file jfb-17-00174-s001.zip › jfb-4171181-supplementary.pdf]

Table S1. Dimensional parameters of PCL@S53P4 scaffolds

		1	2	3	Average	Std. Dev.
PCL	Weight(g)	0.4271	0.421	0.4288	0.4256	0.0033
	Diameter(mm)	14.88	15	14.98	14.9533	0.0525
	Height (mm)	4.38	4.06	4.3	4.2467	0.1360
5%PCL@S53P4	Weight(g)	0.4445	0.4513	0.3937	0.4298	0.0257
	Diameter(mm)	15.12	14.88	14.58	14.8600	0.2209
	Height (mm)	4.32	4.2	3.84	4.1200	0.2040
10%PCL@S53P4	Weight(g)	0.4374	0.3969	0.4334	0.4226	0.0182
	Diameter(mm)	14.78	15.32	15.08	15.0600	0.2209
	Height (mm)	4.32	4.36	4.42	4.3667	0.0411
20%PCL@S53P4	Weight(g)	0.4062	0.4077	0.4044	0.4061	0.0013
	Diameter(mm)	15.04	14.68	14.92	14.8800	0.1497
	Height (mm)	4.38	4.58	4.08	4.3467	0.2055

Table S2. *In Vitro* Degradation of PCL@S53P4 Scaffolds

	Initial Mass (g)	Final Mass (g)	Degradation Rate (%)	Average Degradation Rate (%)
PCL	0.4288	0.4275	0. 3032	0. 3996
	0.4271	0.4250	0. 4917	
	0.4210	0.4193	0. 4038	
5%PCL@S53P4	0.4513	0.4477	0. 7977	0. 7364
	0.4445	0.4415	0. 6749	
	0.3937	0.3908	0. 7366	
10%PCL@S53P4	0.4374	0.4338	0. 8230	0. 7577
	0.4334	0.4305	0. 6691	
	0.3969	0.3938	0. 7811	
20%PCL@S53P4	0.4077	0.4019	1.4226	1.4200
	0.4062	0.4004	1.4279	
	0.4044	0.3987	1.4095	

Table S3. Co-culture of Scaffolds with *S. aureus*

S. aureus	PCL	5%S53P4@PCL	10%S53P4@PCL	20%S53P4@PCL	EXO-APTs-20%S53P4@PCL
OD600-1	0.165	0.138	0.120	0.073	0.026
Inhibition Rate-1（%）		16.3636	27.2727	55.7576	84.2424
OD600-2	0.162	0.117	0.096	0.073	0.022
Inhibition Rate-2（%）		27.7778	40.7407	54.9383	86.4198
OD600-3	0.160	0.125	0.111	0.076	0.056
Inhibition Rate-3（%）		21.8750	30.6250	52.5000	76.8750

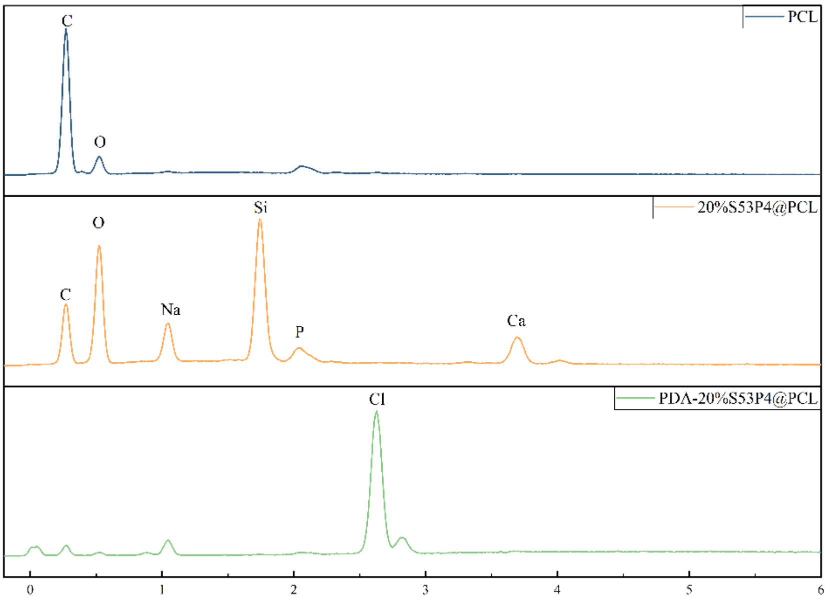

Figure S1. EDS of PDA-20%S53P4@PCL scaffold.

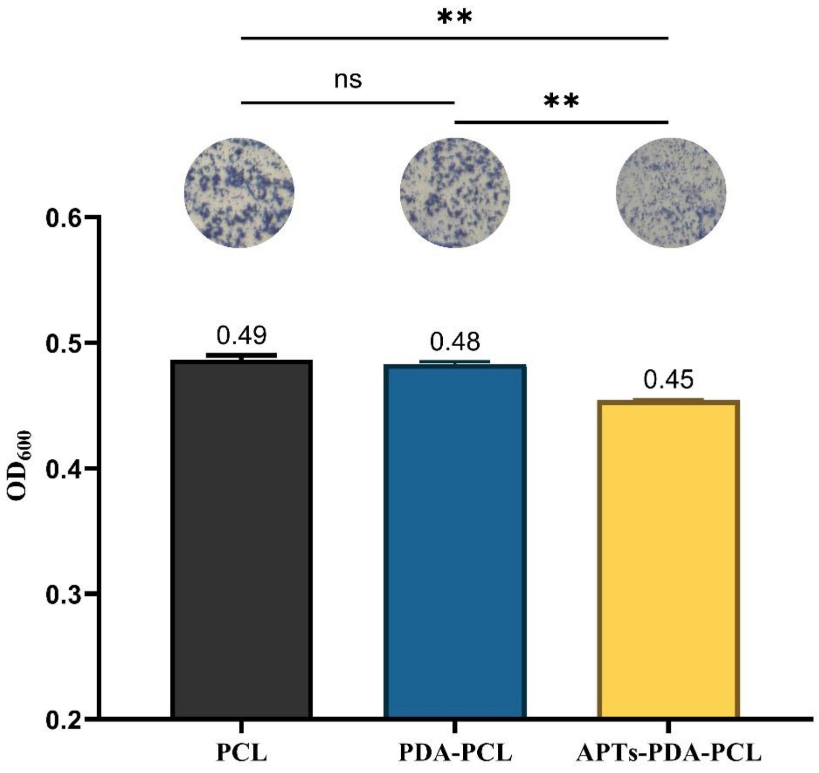

Figure S2. OD_{600} of the remaining suspension was measured after removing the sheets. Data are shown as mean ± SD. n = 3 samples per group. Brown-Forsythe and Welch ANOVA, ** p<0.01.

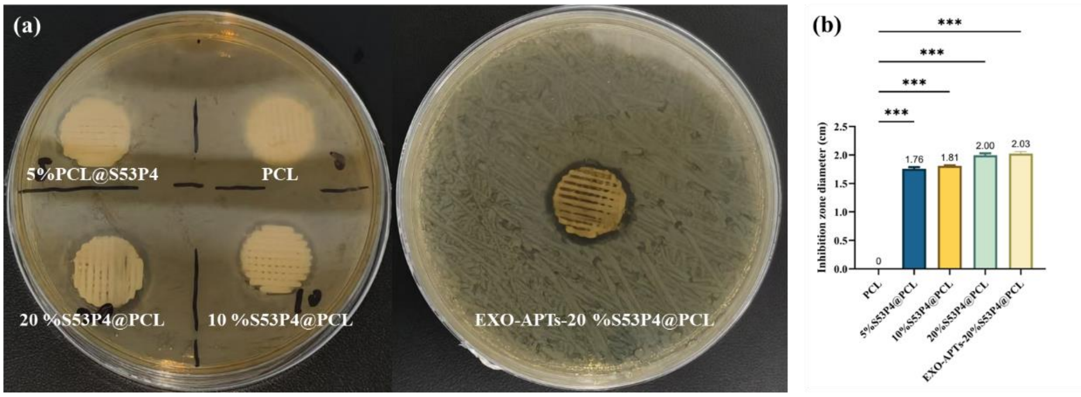

Figure S3. Inhibition zone of scaffolds. (a) Representative images of inhibition zones(b)inhibition zone diameters with statistical analysis. Data are shown as mean ± SD. n = 3 samples per group. One-way ANOVA. ***$p < 0.001$.
